# Pellino Proteins in Viral Immunity and Pathogenesis

**DOI:** 10.3390/v15071422

**Published:** 2023-06-23

**Authors:** Liselotte E. Jensen

**Affiliations:** Department of Microbiology, Immunology and Inflammation, Center for Inflammation and Lung Research, Temple University Lewis Katz School of Medicine, Philadelphia, PA 19140, USA; liselotte.jensen@temple.edu

**Keywords:** ubiquitin, ligase, Pellino, peli, immunity, immune evasion, inflammation, innate immunity, Toll-like receptor, virus

## Abstract

Pellino proteins are a family of evolutionarily conserved ubiquitin ligases involved in intracellular signaling in a wide range of cell types. They are essential for microbe detection and the initiation of innate and adaptive immune responses. Some viruses specifically target the Pellino proteins as part of their immune evasion strategies. Through studies of mouse models of viral infections in the central nervous system, heart, lungs, and skin, the Pellino proteins have been linked to both beneficial and detrimental immune responses. Only in recent years have some of the involved mechanisms been identified. The objective of this review is to highlight the many diverse aspects of viral immunity and pathogenesis that the Pellino proteins have been associated with, in order to promote further research into their functions. After a brief introduction to the cellular signaling mechanisms involving Pellino proteins, their physiological roles in the initiation of immune responses, pathogenesis through excess inflammation, immune regulation, and cell death are presented. Known viral immune evasion strategies are also described. Throughout, areas that require more in-depth investigation are identified. Future research into the functions of the Pellino protein family may reveal fundamental insights into how our immune system works. Such knowledge may be leveraged in the fight against viral infections and their sequala.

## 1. Introduction

Pellino proteins are a group of highly conserved E3 ubiquitin ligases. Ubiquitin is an abundant signaling protein critically involved in numerous cellular processes, ranging from permanent cell differentiation in development to transient immune responses in adulthood. E3 ubiquitin ligases link ubiquitin to target proteins to facilitate their activation, relocalization within the cell, or degradation [1]. As viruses depend on their hosts’ cellular machinery, they extensively interact with such processes, either to benefit from them or to actively suppress them to avoid detection by immune mechanisms. This review is aimed at highlighting the role of the family of Pellino proteins in immunity against viruses, and how these immune responses contribute to pathogenesis. Furthermore, how some viruses are known to evade immune responses involving the Pellino proteins will also be described. 

**Figure 1:**
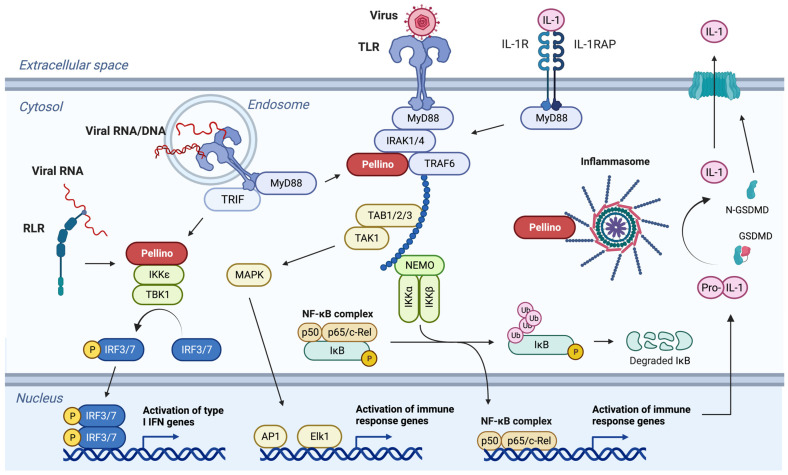
Major signaling pathways regulated by Pellino proteins. Pellino proteins act as intermediate signaling molecules between cytokine, RLR, and TLR in membranes or the cytosol, and transcription factors in the nucleus. Upon IL-1 binding, MyD88 is recruited to the membrane-bound receptor IL-1R1 and IL-1RAP. The IRAK and Pellino proteins are further engaged and promote downstream activation of the IKK (IκB-kinase) complex, which in turn phosphorylates IκB. IκB becomes ubiquitinated and is degraded. This releases the transcription factor NF-κB, which translocates to the nucleus where it activates the expression of pro-inflammatory genes. The pathway can branch to activate MAP kinases essential for activation of the transcription factors AP1 and Elk1. The same signaling cascades can be activated by extracellular TLRs, including TLR2 and TLR4, which may be involved in the recognition of several viral surface proteins. TLR3, TLR7, TLR8, and TLR9, which bind single- and double-stranded RNA and DNA present in endosomes, can activate the same pathways in an MyD88-dependent or -independent manner. The latter requires TRIF and results in interactions between Pellino proteins and the two kinases IKKε and TBK1. Through phosphorylation, this results in the activation of IRFs. The IRFs form homo- and heterodimers that in the nucleus bind to promoter regions of genes encoding type I IFNs. The Pellino•IKKε•TBK1•IRF pathway is also engaged by RLRs such as RIG-I and MDA5, which recognizes viral RNA in the cytosol. These pathways also contribute to activation of the inflammasome. Pellino primes the inflammasome through the ubiquitination of NLRP3 and ASC. *IL1B* is among the many immune response genes activated by NF-κB. The gene transcript is translated into pro-IL-1β, which is activated through cleavage by caspase-1 in the inflammasome. In a similar manner, GSDMD (gasdermin-D) is cleaved. The N-terminal fragment N-GSDMD oligomerizes and forms membrane pores through which IL-1β is released. These pores can also contribute to pyroptotic cell death. The specific mechanisms engaged during viral infection appear to be cell type- and context-dependent (see text for details). The individual signaling cascades and the involvement of specific Pellino proteins are more comprehensively reviewed elsewhere [3,4].

**Figure 2:**
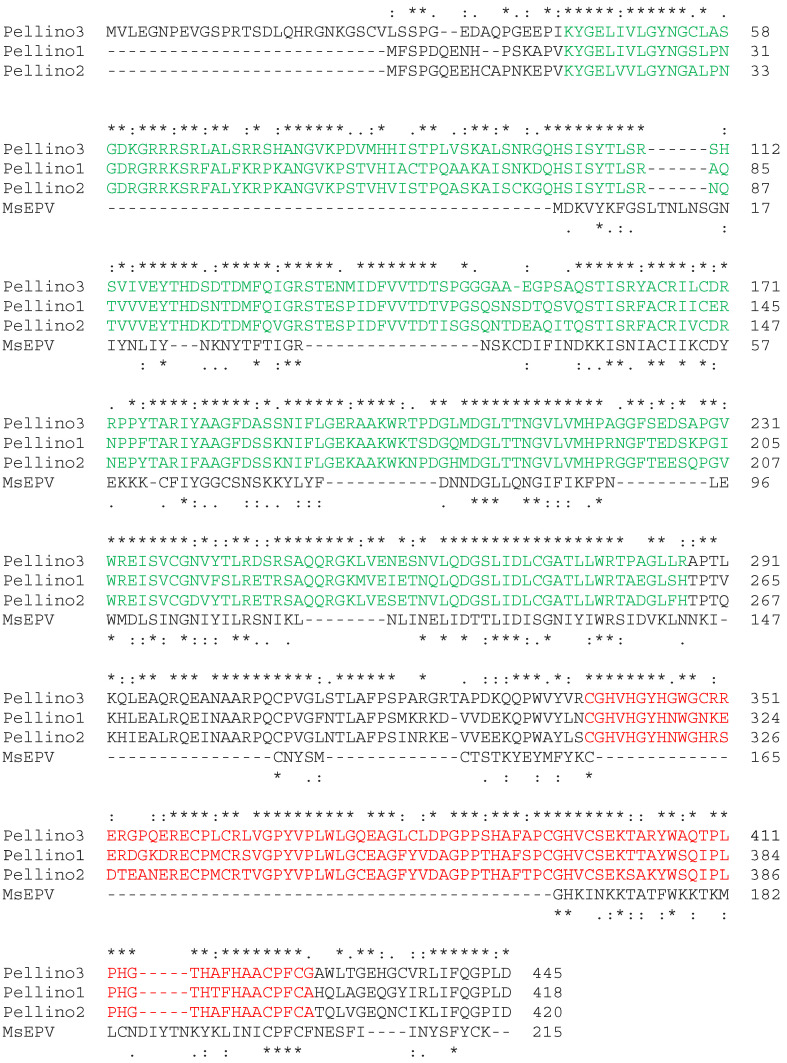
Pellino proteins contain highly conserved functional domains. Human Pellino1, Pellino2, and Pellino3 were aligned with the *Melanoplus sanguinipes* entomopoxvirus (MsEPV) Pellino protein using Clustal Omega at https://www.ebi.ac.uk/Tools/msa/clustalo/ (accessed on 12 May 2023). Stars (*) and dots (: and .) above the alignment indicate, respectively, identical and similar amino acids in the human proteins. Stars (*) and dots (: and .) below the alignment indicate, respectively, identical and similar amino acids between the human proteins and MsEPV Pellino. The FHA domain is shown in green. The RING domain is shown in red.

The first Pellino protein was identified in *Drosophila melanogaster* as part of the Toll•Tube•Pelle signaling cascade essential for dorsal–ventral pattern development in the fly [2]. In vertebrates, the related TLR (Toll-like receptor) MyD88 (myeloid differentiation primary response protein 88) IRAK (interleukin-1 receptor (IL-1R)-associated kinase), and TLR•TRIF (Toll/IL-1R-domain-containing adapter-inducing interferon-β) pathways engage numerous immune mechanisms in myeloid and non-myeloid cells (Figure 1). The many and diverse signaling pathways that the Pellino proteins regulate are comprehensively reviewed elsewhere [3,4]. Chief among these mechanisms is the activation of the ubiquitous nuclear transcription factor NF-κB, MAPK (mitogen-activated protein kinases), and IRF (interferon (IFN) response factors) [3,4]. The ubiquitin ligase activity of the Pellino proteins is facilitated by a RING (really interesting new gene) domain located at the C-terminal end (Figure 2) [5,6]. At the N-terminus is an FHA (forkhead-associated) domain that is involved in interacting with phosphorylated IRAK1 (Figure 2) [7].

In addition to Drosophila, Pellino has also been identified in nematodes, including *Caenorhabditis elegans* and *Schistosoma mansoni* [8,9], as well as in several crustacean species [10,11,12,13]. In mammals, the three *PELI* genes encode the proteins Pellino1, Pellino2, and Pellino3 (Figure 2) [13]. The degree of sequence conservation is striking with, for example, only a single amino acid substitution between human and mouse Pellino1. Despite the high degree of similarity between the invertebrate and vertebrate Pellino proteins, the knockout of individual *Peli* genes in mice does not lead to apparent developmental defects [14,15,16,17,18]. Thus, the Pellino proteins appear to have, at least in part, evolved as part of the more elaborate immune system in vertebrates.

## 2. Initiation of Immune Responses

A crucial role of the Pellino proteins is to activate the expressions of genes involved in immune responses. In the context of viral infections, the research focus has so far been on innate immune mechanisms and inflammation. The innate immune response involves the direct detection of viruses independent of adaptive immunity. The cell membrane- and endosome-associated TLRs bind molecular patterns and proteins associated with microbes [19,20]. In a similar manner, the RIG-I (retinoic acid-inducible gene I)-like receptors (RLRs) are a group of cytosolic sensors of viruses, for example [21]. Early mechanistic studies in cell culture systems established the role of Pellino proteins as intermediate signaling molecules from TLRs (Figure 1). In human bronchial epithelial cells, Pellino1 is essential for production of the inflammatory cytokine CXCL8, also known as IL-8, in response to double-stranded RNA and rhinovirus minor group serotype 1B (RV-1B), a common human pathogen [22]. The same study found that Pellino1 is not required for production of the antiviral IFNβ, a type I IFN [22]. In contrast, Pellino1 appears to be critical for IFNβ expression in myeloid cells and embryonic fibroblasts in response to double-stranded RNA and Sendai virus [23,24]. Independently, it has been shown that Pellino3 is an essential intermediate between RIG-I detection of the vesicular stomatitis virus (VSV) and cytokine production, including type I IFN (Figure 1), in bone-marrow-derived macrophages [25]. This may suggest differential roles for Pellino proteins in antiviral immunity in different cell types. In fact, in vivo studies using viruses with tropism for different organs and cell types have indeed revealed distinct outcomes for infections.

In a mouse model of the herpes simplex virus-1 (HSV-1) skin infection, Pellino1-deficient mice develop larger lesions and have a higher viral load in affected skin [16]. However, in mouse models of lung infections with rhinovirus RV1B and influenza A virus (IAV), Pellino1 does not appear to be essential for immunity, as viral loads were found to be the same in both Pellino1 knockout and wild-type mice [26]. In addition to the viral tropisms and separate roles of Pellino1 in different cell types, the difficulty in demonstrating involvement of Pellino in innate antiviral immunity may also be due to redundancies in immune mechanisms to ensure adequate protection, even in the presence of viral immune evasion strategies.

**Figure 3:**
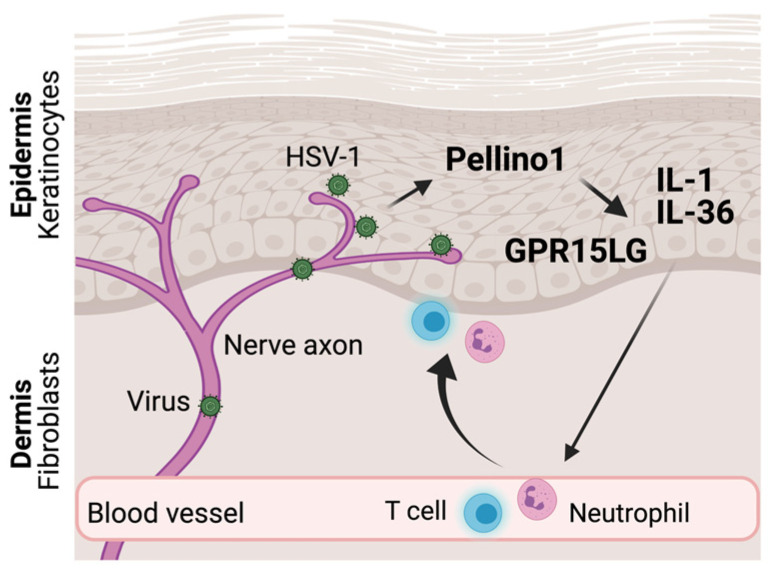
Pellino proteins promote recruitment of leukocytes into tissues. HSV-1 establishes lifelong latent infections in neurons. Upon reactivation, HSV-1 reemerges from axons innervating the skin (shown) or mucosa. Viral replication in epithelial keratinocytes promotes expression of several pro-inflammatory cytokines in a Pellino1-dependent manner. While IL-1 and IL-36 facilitate recruitment of neutrophils from the circulation, GPR15LG is chemotactic to T cells expressing GPR15.

In addition to IFN-regulated viral restriction mechanisms that may limit viral replication directly in the infected cells, recruitment of leukocytes is an essential component of immunity in vivo. As mentioned above, CXCL8 is expressed in a Pellino1-dependent manner in contexts relevant to viral infections [22]. CXCL8 is chemotactic specifically to neutrophils and is regulated by, for example, IL-1 and IL-36 [27,28]. Mice do not have CXCL8, and often utilize CXCL1 and CXCL2 for tissue recruitment of neutrophils. Both of these cytokines are also regulated by IL-1 and IL-36 [27,28]. During HSV-1 skin infection in mice, neutrophil recruitment to naïve infection sites is delayed in Pellino1-deficient animals [16]. This correlates with reduced levels of IL-1 and IL-36 (Figure 3) [16]. However, the exact role of neutrophils in this model is unclear, as it has independently been shown that depletion of neutrophils does not affect viral loads in the infected skin [29,30]. A similar situation exists in the female reproductive system. While IL-36 promotes neutrophil recruitment into HSV-2-infected vaginal mucosal sites, the antiviral functions of IL-36 are independent of neutrophils [31]. The Pellino proteins are expressed in both male and female reproductive organs, but their possible involvement in immunity against sexually transmitted diseases, for example, has not been examined.

An essential bone-marrow-derived cell type for antiviral immunity is T cells. In VSV-infected macrophages, Pellino3 promotes the expression of CXCL10, previously known as IP-10 [25], a well-established regulator of T cell recruitment, priming, and development [32,33]. A more recently identified modulator of T cells is GPR15LG, formerly known as *2610528A11Rik* in mice and *C10orf99* in humans. GPR15LG is the ligand for GPR15 on T cells and acts as a chemoattractant [34,35]. It is an important regulator of inflammation in the skin [36], and its reduced expression in Pellino1 knockout mice may explain delayed T cell recruitment during HSV-1 skin infections (Figure 3) [16]. This, in turn, may explain why HSV-1 replicates and disseminates better in Pellino1-deficient mice compared to wild-type mice [16]. If GPR15LG is important for antiviral immunity in other tissues frequently infected by viruses remains unknown.

The inflammasome is a multimeric protein complex essential for the proteolytic activation of IL-1β [21]. It is extensively regulated through ubiquitination [37]. *IL1B* is among the immune response genes activated during the early detection of viral infection (Figure 1). The protein IL-1β is initially synthesized as an intracellular pro-protein without a signal peptide for extracellular export (reviewed in [38]). For example, upon RLR or TLR engagement (Figure 1), Pellino1 and Pellino2 can ubiquitinate ASC (adaptor molecule apoptosis-associated Speck-like protein containing a CARD (caspase activation and recruitment domain)) and NLRP3 (nucleotide-binding domain, leucine-rich-containing family, pyrin domain-containing-3) [14,39,40,41]. This ubiquitination primes formation of the NLRP3•ASC inflammasome to which inflammatory caspases such as caspase-1 are also recruited (Figure 1). Caspase-1 cleaves pro-IL-1β and Gasdermin-D into their mature forms. The N-terminal domain of the latter forms large pores in the cell membrane through which the cleaved IL-1β is released (Figure 1, reviewed in [38]). While the Pellino proteins have been shown to have important beneficial and detrimental roles during viral infections, as described above and below, the involvement of the Pellino-to-inflammasome signal during these viral infections remains largely unexplored.

## 3. Viral Immune Evasion

Viruses extensively deploy immune evasion strategies to avoid detection by innate sensors and prevent activation of the inflammasome, reviewed in detail elsewhere [38,42,43,44,45,46,47,48]. During evolution, apparent redundancies have emerged as fail safes to ensure protective immunity even in the presence of highly effective microbial immune evasion mechanisms. Consequently, deletion of one or more of the inflammasome components from the mouse genome may have a limited, if any, effect upon infection outcomes. For example, mice deficient in the two inflammasome executioner proteases, caspase-1 and caspase-11, appear to develop normal protective immunity against HSV-1, as evidenced by their viral dissemination being on par with wild-type mice [49]. While several viral immune evasion mechanisms have been identified involving Pellino-activated NLRP3 and ASC [38,42,43,44,45], specific viral targeting of one or more of the Pellino proteins in these processes has not been examined. However, an unrelated direct viral immune evasion mechanism has been identified in the *Melanoplus sanguinipes* entomopoxvirus (MsEPV) [50].

**Figure 4:**
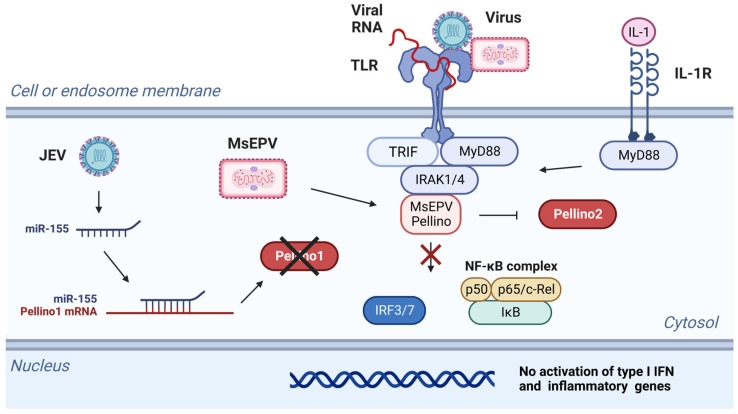
Viruses can block Pellino1 activity through different mechanisms. MsEPV encodes a Pellino protein that binds to IRAK but fails to engage downstream signaling. This prevents the production of type I IFNs and inflammatory cytokines. JEV enhances expression of the host miR-155, which in turn suppresses expression of Pellino1 and inflammatory genes. Black X indicates that the Pellino1 protein is not synthesized. Red X indicates that the pathway cannot take place.

Poxviridae is a large family of double-stranded DNA viruses that can infect vertebrate and arthropod species. MsEPV belongs to the Entomopoxvirinae subfamily and infects, for example, the North American migratory grasshopper *Melanoplus sanguinipes* [51]. The MsEPV genome includes 267 methionine-initiated open reading frames greater than 60 amino acids [51]. One of these genes encodes a ubiquitin-like protein [51], while another translates into a Pellino homolog with an overall 15.6% sequence identity to human Pellino2 and 27.6% identity within the FHA domain (Figure 2) [50,51]. While the FHA domain is present in MsEPV Pellino, the ubiquitin ligase activity associated RING domain is truncated in MsEPV Pellino (Figure 2) [50]. Consequently, MsEPV Pellino associates with IRAK but fails to engage downstream signaling events (Figure 4) [50]. The overexpression of MsEPV Pellino in both insect and human cells causes inhibition of TLR-mediated NF-κB or NF-κB-like activity. As such, MsEPV Pellino appears to act as an active strategy to prevent the initiation of immune responses within the infected cells (Figure 4) [50]. The relatively weak sequence similarity between MsEPV and Pellino2 (Figure 2) leaves open the possibility that other viral-encoded Pellino-like proteins remain to be identified [50].

A more indirect mechanism of immune evasion may be employed by the Japanese encephalitis virus (JEV) [52]. During JEV infection of microglia, miR-155 is upregulated (Figure 4). By complementary base-pairing, microRNAs silence mRNAs through degradation or suppression of translation. The miR-155 prevents expression of Pellino1 [52,53], and in the microglia-JEV system, this results in the reduced expression of proinflammatory genes (Figure 4) [52]. If other microRNAs are involved in regulating Pellino1, Pellino2, or Pellino3, they have not yet been examined.

## 4. Excess Inflammation and Pathogenesis

**Figure 5:**
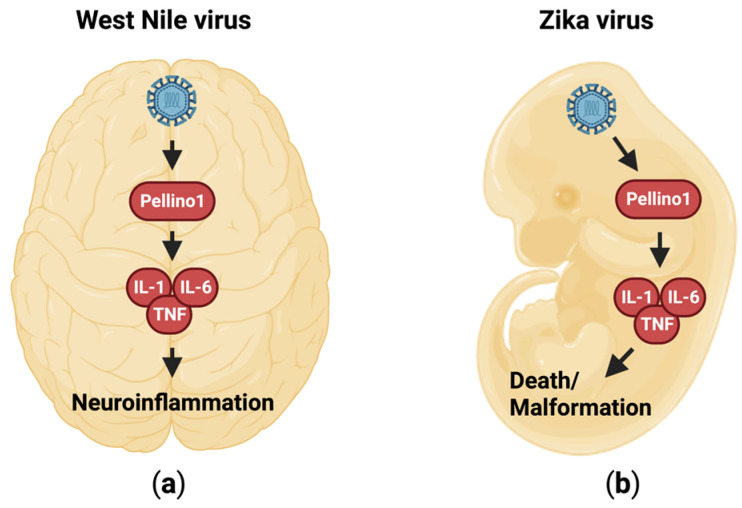
Flaviviruses promote neuroinflammation through a Pellino1-dependent mechanism. (**a**) Pellino1 promotes IL-1, IL-6, and TNF expression and neuroinflammation in response to infection with WNV in the adult brain. (**b**) During embryonic ZV infection, Pellino1 drives the expressions of IL-1, IL-6, and TNF. This results in inflammation that causes malformations and death.

Inflammation is essential for recruiting immune cells to infected tissues, as described above. The immune cells are involved in killing infected cells, clearing cellular debris, and promoting tissue regeneration. However, an exuberant inflammatory response that is too potent or prolonged can damage the surrounding uninfected tissue, and thus exacerbate and/or prolong disease. The central nervous system is especially prone to damage from inflammation. The two flaviviruses, West Nile virus (WNV) and Zika virus (ZV), trigger inflammation that is Pellino1-dependent (Figure 5) [18,54]. WNV enters the brain by crossing the blood–brain barrier, and promotes meningitis and encephalitis [18]. The onset and progression of this inflammation is attenuated in Pellino1-deficient mice (Figure 5a) [18]. In a similar Pellino1-dependent manner, ZV enters and crosses the placenta [54], and the concomitant inflammation results in fetal demise (Figure 5b) [54]. In both models, innate inflammatory markers such as IL-1β, IL-6, and TNF are reduced in Pellino1-deficient mice (Figure 5) [18,54]. The entry and replication of both viruses in several cell types, including macrophages, microglia, and neurons, is Pellino1-dependent through an as yet unknown mechanism [18,54]. Thus, the targeted inhibition of Pellino1 function may have dual preventive or therapeutic applications through both reduction of inflammation and restriction of viral replication.

Viral infections can exacerbate many inflammatory diseases. Chronic obstructive pulmonary disease (COPD) is the third-leading cause of death worldwide, and is characterized by progressively worsening lung inflammation. Both bacterial and viral infections of the airways aggravate the disease. When infected with the rhinovirus RV-1B, primary bronchial epithelial cells from patients with COPD produce elevated cytokine levels compared to cells from healthy control subjects [55,56]. Pellino1 is specifically upregulated upon RV-1B infection in cells from COPD patients [55]. While this increase in Pellino1 production may contribute to the observed elevated cytokine expression in infected COPD cells, its potential role in exacerbating COPD during upper respiratory viral infections remains undetermined.

## 5. Immunoregulatory Functions

Since excessive inflammatory responses can result in tissue damage if they are not tightly controlled, numerous regulatory mechanisms are in place to curb the degree and duration of the involved pathways, including those engaged by Pellino proteins (Table 1). Pellino3 may have evolved, at least in part, to regulate certain inflammatory signaling cascades. Through respectively poly- and mono-ubiquitination, Pellino3 modifies TRAF6 (tumor necrosis factor receptor-associated factor-6), TRAF3, and TANK (TRAF family member-associated NF-κB activator) [15,57,58]. These ubiquitination events disrupt interactions with downstream signaling factors TBK1 (TANK-binding kinase 1, an activator of IRF3), IRF3, and IRF7, and thus diminish IFNβ induction [15,57]. In the absence of these mechanisms, a stronger immune response is engaged. Consequently, Pellino3-deficient mice exhibit less severe disease, including lower viral loads during at least encephalomyocarditis virus (EMCV) infections [15].

Similarly, Pellino1 promotes degradation of TRAF3 [59] and suppression of type I IFN in microglia cells [60]. Thus, Pellino1 knockout mice have a more potent antiviral immune response against VSV, including higher levels of type I IFNs and TNF, and better viral clearance than wild-type mice [60]. Pellino1-deficient mice also have increased inflammation and production of TNF and IL-6 during infections with RV1B and IAV [26]. However, the specific mechanism involved has not been investigated.

Pellino1 is a known regulator of T cell polarization through the ubiquitination of c-Rel [53,61]. c-Rel is a member of the NF-κB family of transcription factors. It is predominantly expressed in B and T cell lineages and is essential for proliferation and differentiation [65]. During T cell development, Pellino1 promotes the degradation of c-Rel [61]; however, the expression of Pellino1 is repressed by miR-155 [53]. This regulatory activity creates a fine-tuned system that is essential for preventing the development of self-reactive T cells. Consequently, upon appropriate non-microbial challenge, Pellino1-deficient mice develop autoimmunity [61,66]. Numerous viruses have been linked to the development of autoimmunity [67,68], but how the Pellino1•miR-155 system may be involved in these processes has not been examined.

**Figure 6:**
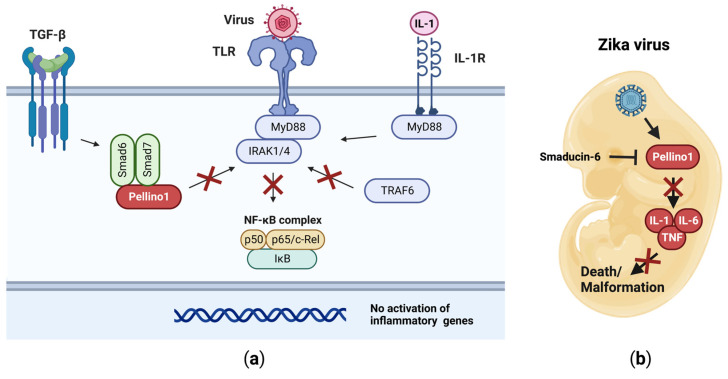
Pellino1 is inhibited by Smad6 and Smad7. (**a**) Following activation via TGF-β, Smad6 and Smad7 bind to Pellino1. This prevents Pellino1•IRAK interactions, and thus downstream signaling events are not engaged. (**b**) Smaducin-6, a peptide based on the Smad6 sequence, inhibits Pellino1 during ZV infections. This results in attenuated IL-1-, IL-6-, and TNF-dependent inflammation and death in the developing embryos. Red Xs indicate pathways that cannot take place.

Cellular responses take place in highly complex milieus. TGF-β (transforming growth factor) is a cytokine with anti-inflammatory properties through, for example, Smad6 and Smad7 (suppressor of mothers against decapentaplegic) [69]. Upon their induction, Smad6 and Smad7 cooperate to suppress IL-1 and TLR signaling [62,63]. Smad6 associates with the FHA domain in Pellino1, and thus disrupts the interface essential for binding to IRAK1 (Figure 6a) [62]. Smad7 binds to the region between the FHA and RING domains in Pellino1 (Figure 2) [63]. These interactions prevent the assembly of the MyD88•IRAK1•Pellino1•TRAF6 signalosome, and thus the activation of the downstream pro-inflammatory signaling cascades, including the NF-κB pathway (Figure 6a) [62]. This mechanism has been harnessed to prevent developmental defects in mouse embryos during infection with ZV using a peptide (Smaducin-6) derived from Smad6 (Figure 6b) [54]. Whether this approach will translate into humans and other viral pathologies involving Pellino1-dependent tissue damage remains to be determined.

IDO (indoleamine 2,3-dioxygenase) acts as an immunomodulator through the catabolism of tryptophane [64]. The enzyme suppresses inflammatory responses and promotes tolerance by shifting the balance between effector T cells and regulatory T cells towards the latter [64]. During viral infections, IDO expression is induced by type I and type III IFNs [64]. Experiments involving the IDO inhibitor 1-methyl tryptophan (1MT) suggest that IDO suppresses Pellino1 expression in macrophages, and that this contributes to restrained production of inflammatory cytokines during influenza infections [70]. Hence, the regulation of at least Pellino1 is intricate (Table 1) during viral infections, and its specific role in promoting beneficial and detrimental inflammatory responses requires further investigation.

## 6. Regulation of Cell Death Mechanisms

Cell death impacts many diverse cellular and physiological functions during viral infections. There are currently 22 defined types of cell death [71]; of these, Pellino proteins are known to regulate 4. One of these is pyroptosis, which is associated with activation of the inflammasome and extracellular release of IL-1β, described above (Figure 1). The formation of N-GSDMD pores in the cell membrane can result in loss of membrane potential, leading to cell death through pyroptosis [72,73,74,75,76,77]. Since both Pellino1 and Pellino2 are involved in activation of the inflammasome [14,39,40,41], they may be determinants of the outcome of viral infections through pyroptosis. While mice deficient in caspase-1 and caspase-11 appear fully competent at controlling HSV-1 and exhibit no more severe disease than wild-type mice [49], other viruses need to be examined to define the beneficial and detrimental effects of pyroptosis on inflammation, tissue damage, and viral restriction.

**Figure 7:**
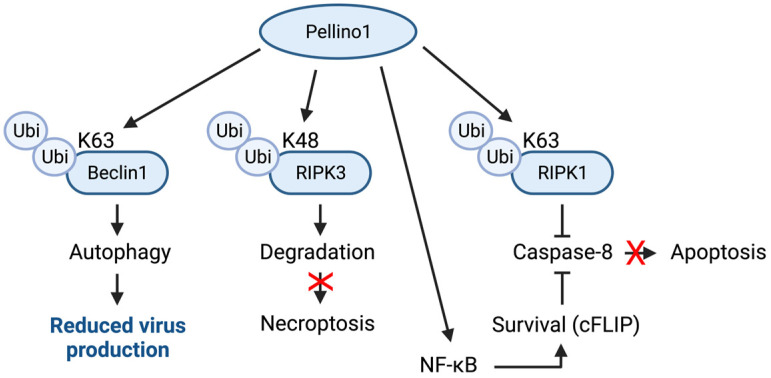
Cell death pathways are modified by Pellino1. Pellino1 can suppress necroptosis through K48 ubiquitination of RIPK3. Pellino1-mediated induction of survival genes, such as cFLIP, and K63 ubiquitination of RIPK1 inhibit caspase-8 activation and thus, apoptosis. Through K63 ubiquitination, Pellino1 activates Beclin1 and autophagy. Red Xs indicate pathways that cannot take place.

Programmed cell death pathways such as apoptosis and necroptosis can act as antiviral mechanisms, but viruses extensively suppress them through their survival strategies [44,78]. The Pellino proteins appear to regulate these pathways in many ways. Pellino1 can reduce necroptosis in keratinocytes through K48-linked ubiquitin-mediated degradation of RIPK3 (receptor-interacting serine/threonine-protein kinase) (Figure 7) [79]. Pellino1 may also suppress apoptosis (Figure 7). This can be achieved through at least two different mechanisms. K63-ubiqutination of RIPK1 prevents interaction with caspase-8, while NF-κB-elicited expression of survival genes, e.g., cFLIP (cellular Fas-associated death domain-like IL-1β-converting enzyme-inhibitory protein), provides an alternative approach to preventing caspase-8 activation [80], and thus downstream apoptosis (Figure 7). It should be noted that how these mechanisms affect the outcomes of viral infections in different cell types has not been studied.

An alternative viral restriction approach is autophagy [81]. The HIV Tat protein promotes autophagy in endothelial cells [82]. In this system, Pellino1 activates Beclin1, an activator of autophagy [83], through K63-ubiquitination (Figure 7); this may be a contributing factor to blood–brain barrier dysfunction in HIV patients [82]. How this mechanism may impact viral replication needs to be examined. It also remains to be determined if Pellino2 and Pellino3 have similar or diverging functions.

## 7. Conclusions

The involvement of Pellino proteins in immunity and pathogenesis during viral infections is multifaceted. While having essential protective functions, they may also contribute to pathologies. Prophylactic and therapeutic approaches targeting these mechanisms may be possible; however, such strategies must be carefully evaluated for both their beneficial and detrimental effects. The already known Pellino-regulated pathways and mechanisms described in this review cannot necessarily be directly extrapolated from one system to another, due to the unique complexity of individual organs and each viral pathogen having specific immune evasion strategies. Thus, numerous opportunities exist to not only elucidate the roles of Pellino-mediated ubiquitination in immune and disease processes, but likely also identify new viral approaches to modifying ubiquitin-regulated cellular responses. Comprehensive insight into such functions and mechanisms is essential in the fight against current and future pathogenic viruses. Further research should carefully delineate favorable from damaging activities to best prepare us for the next pandemic.

## Figures and Tables

**Table 1:** Regulatory functions of Pellino proteins. Pellino1 and Pellino3 can through ubiquitination promote degradation of direct or indirect downstream signaling factors. Pellino1 itself is regulated at the mRNA and protein levels through the action of inhibitory molecules. These factors act upstream of the Pellino1-activated signaling cascades.

Pellino Protein	Downstream Degraded Target	Upstream Regulator
Pellino3	TRAF6 [15]	
Pellino3	TRAF3 [58]	
Pellino3	TANK [57]	
Pellino1	TRAF3 [59,60]	
Pellino1	c-Rel [61]	miR-155 [53]
Pellino1		Smad6 [62]
Pellino1		Smad7 [63]
Pellino1		IDO [64]
